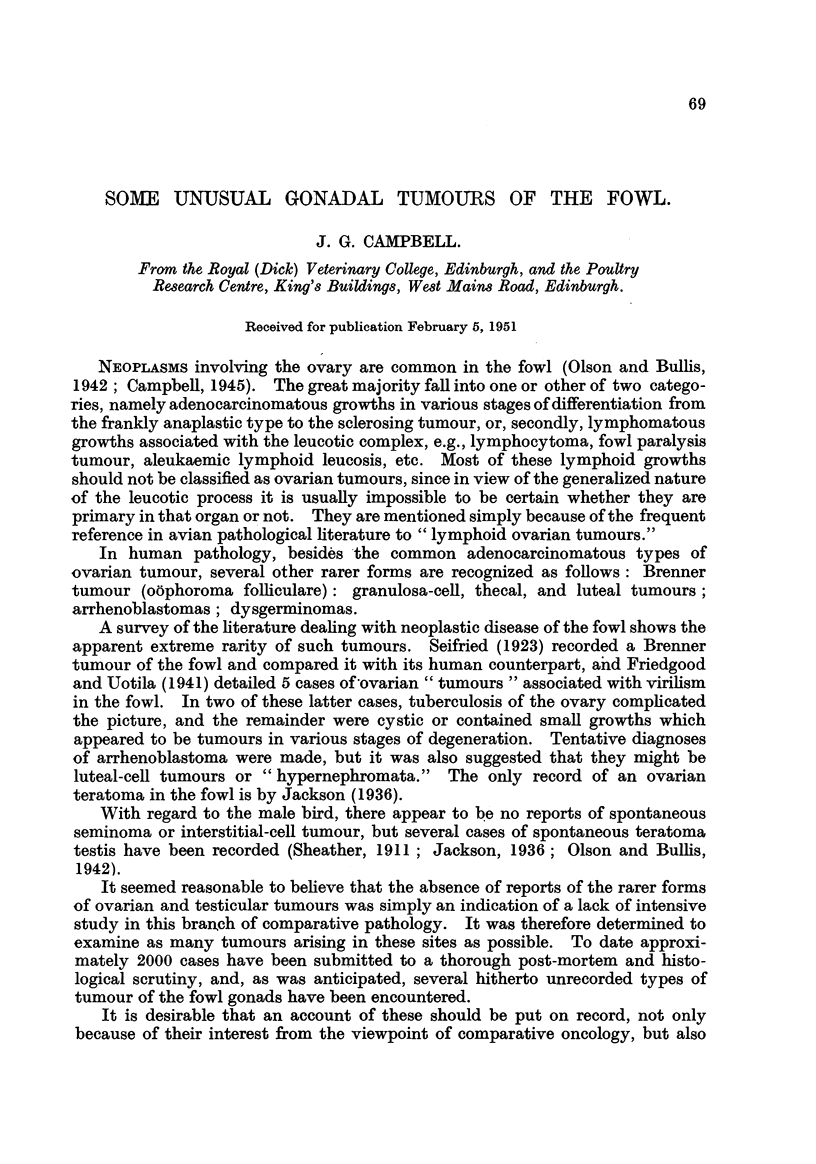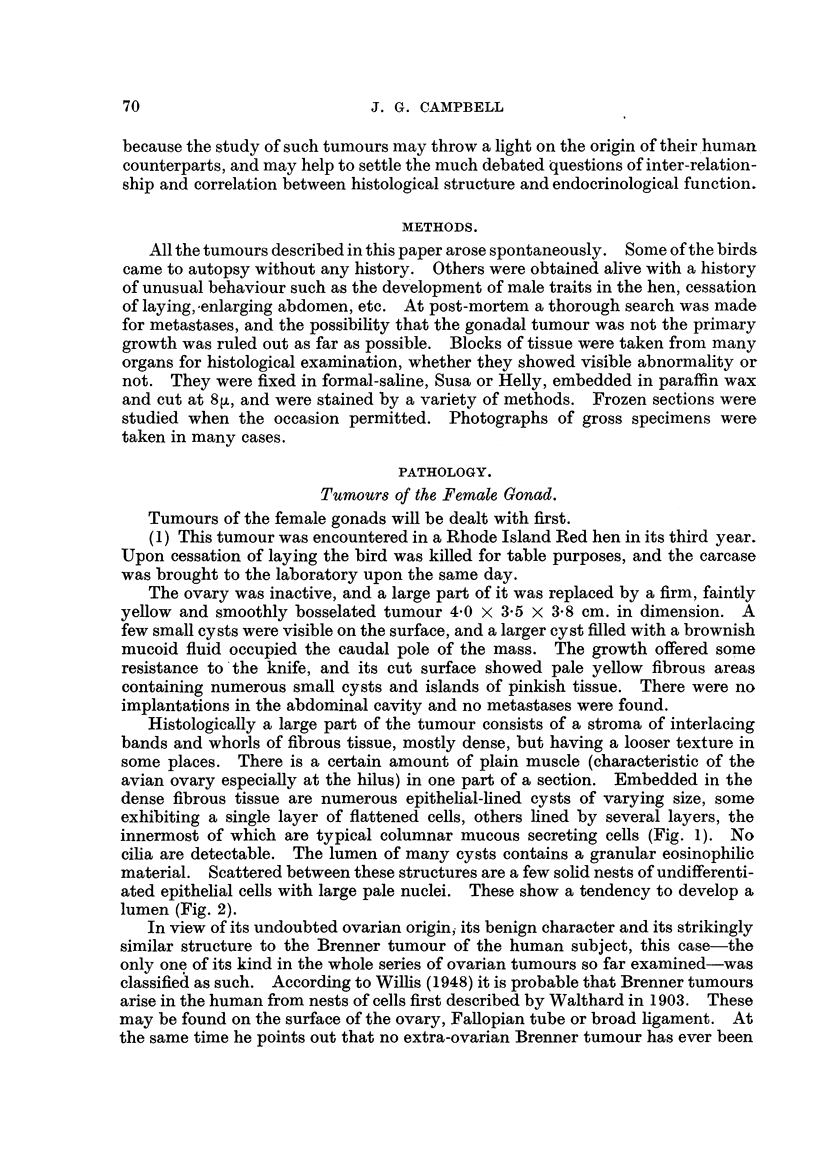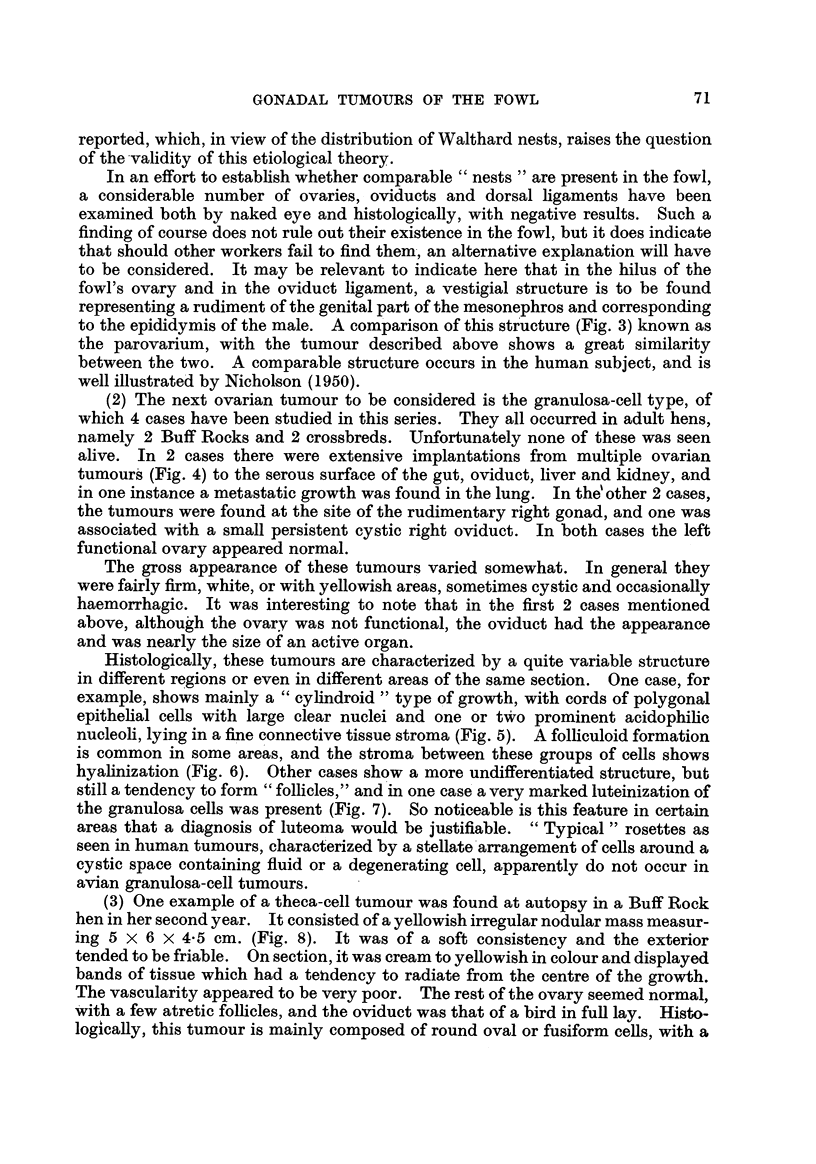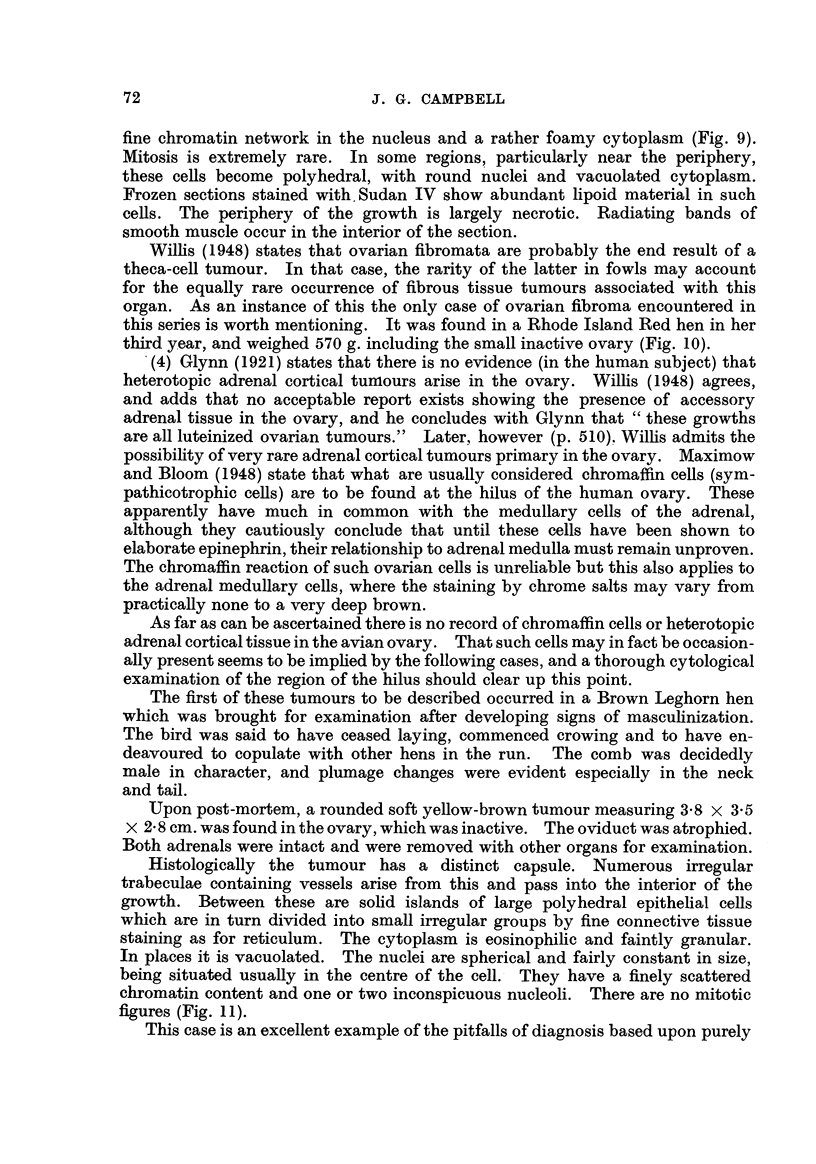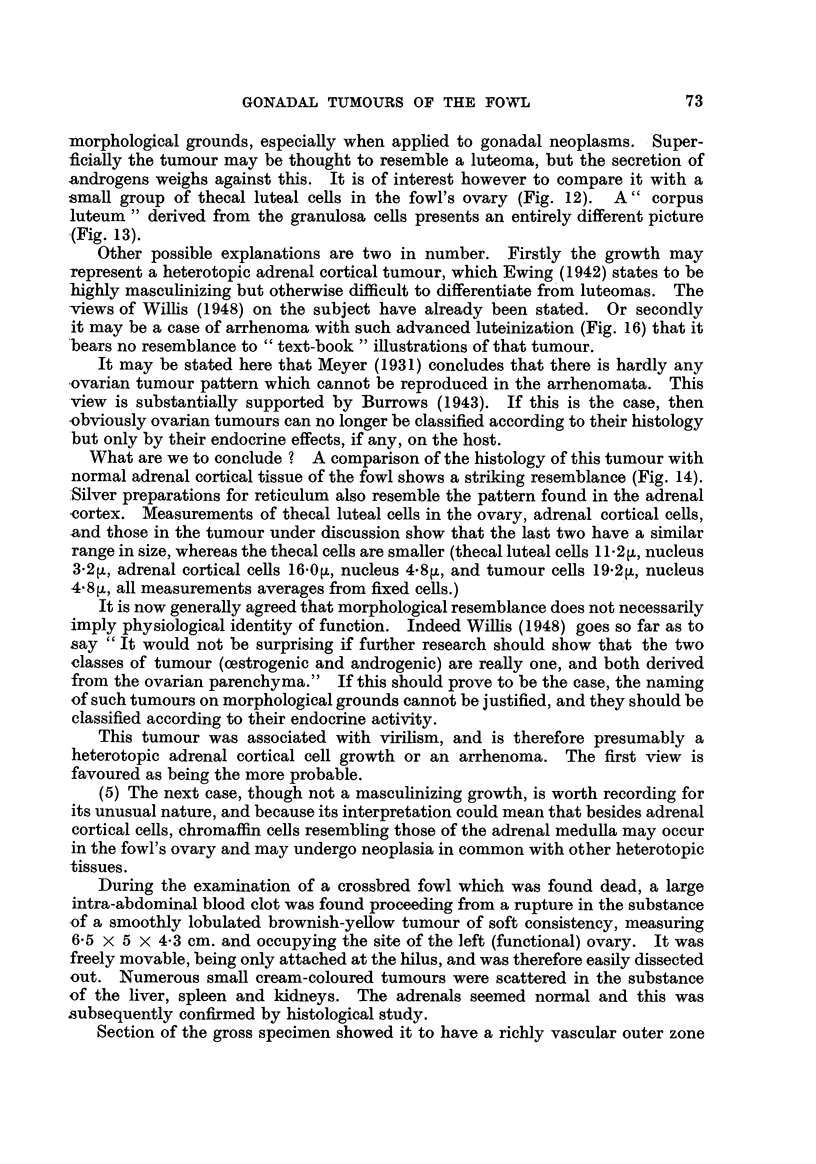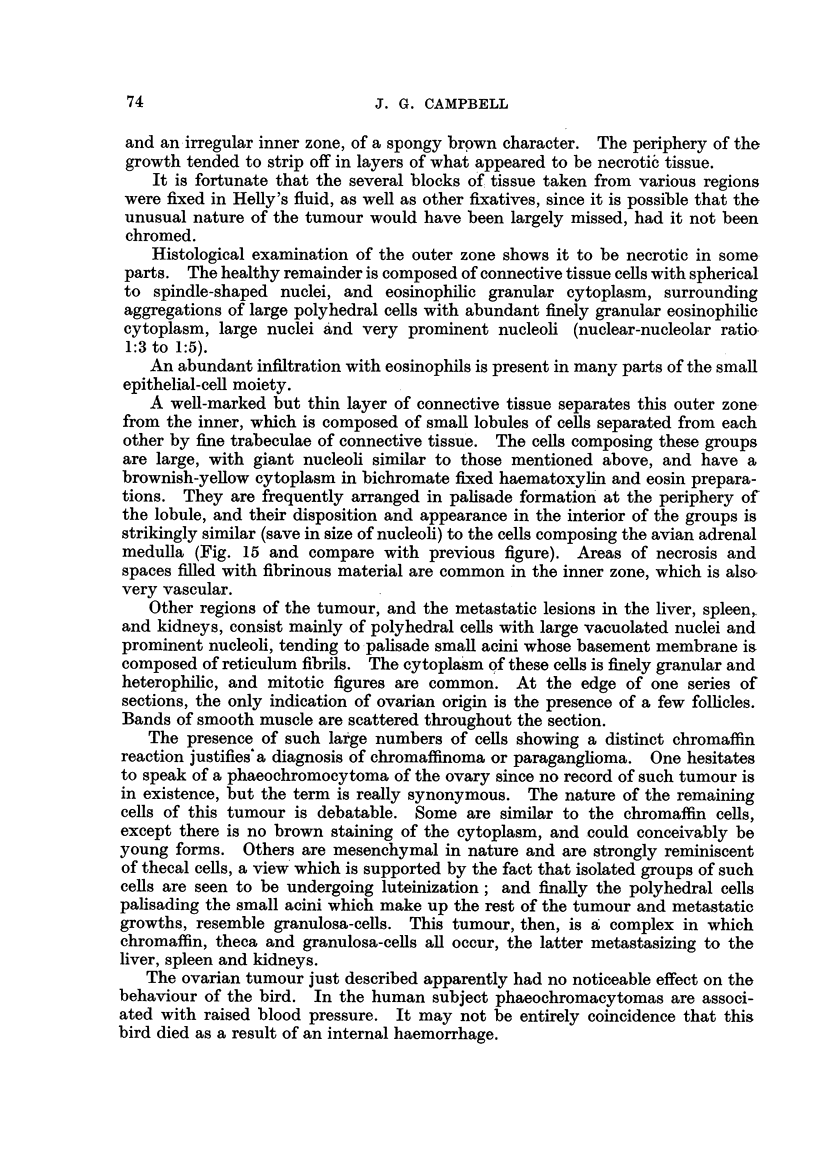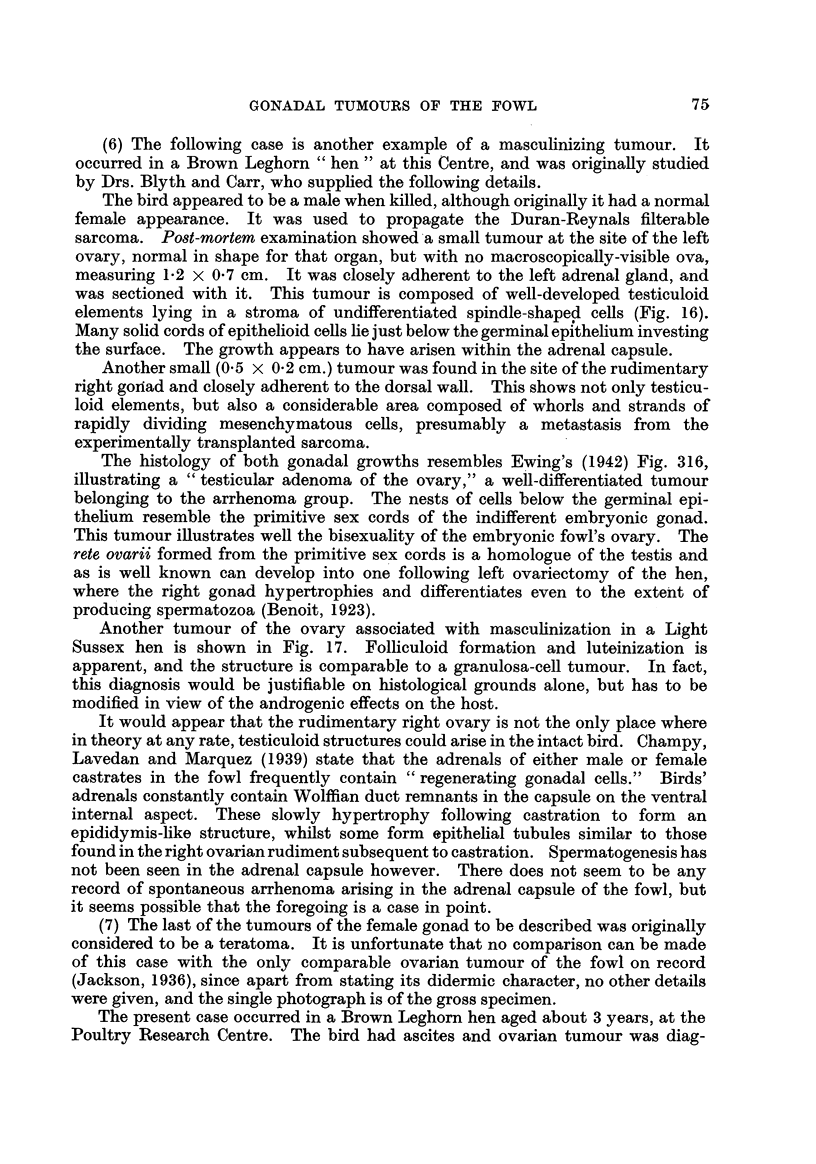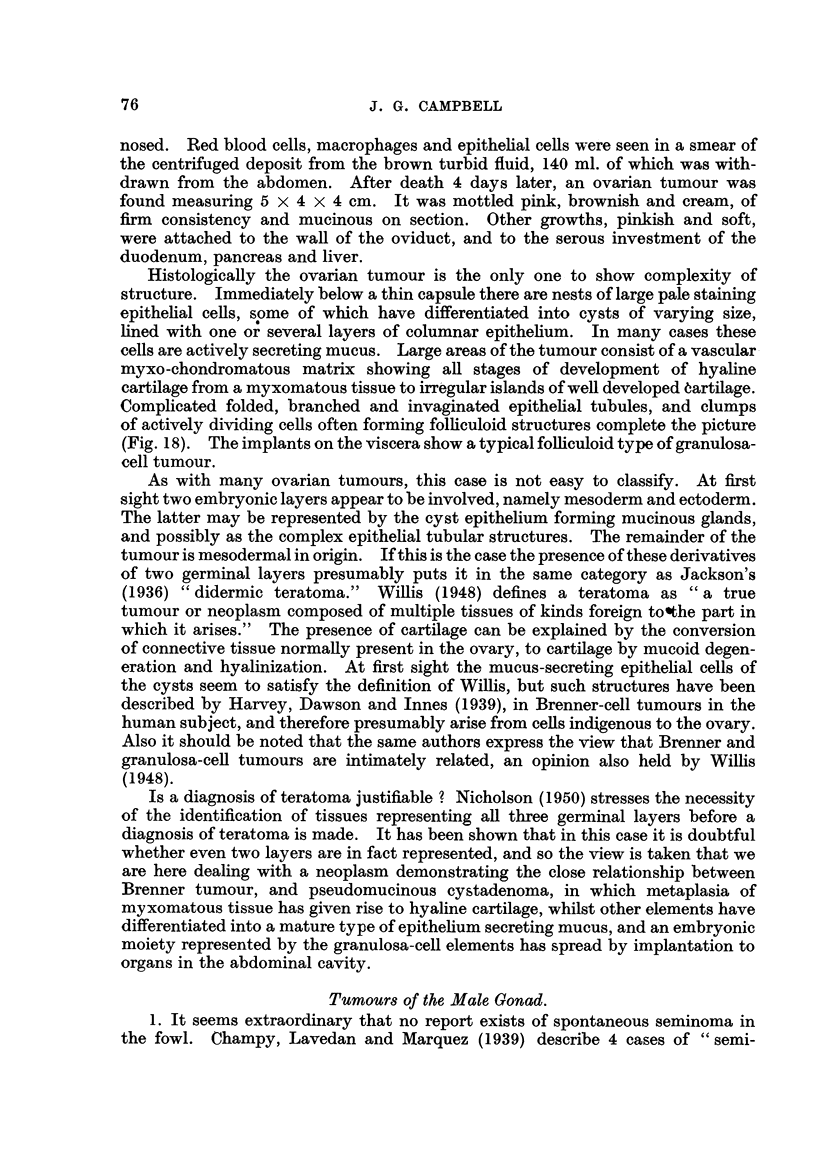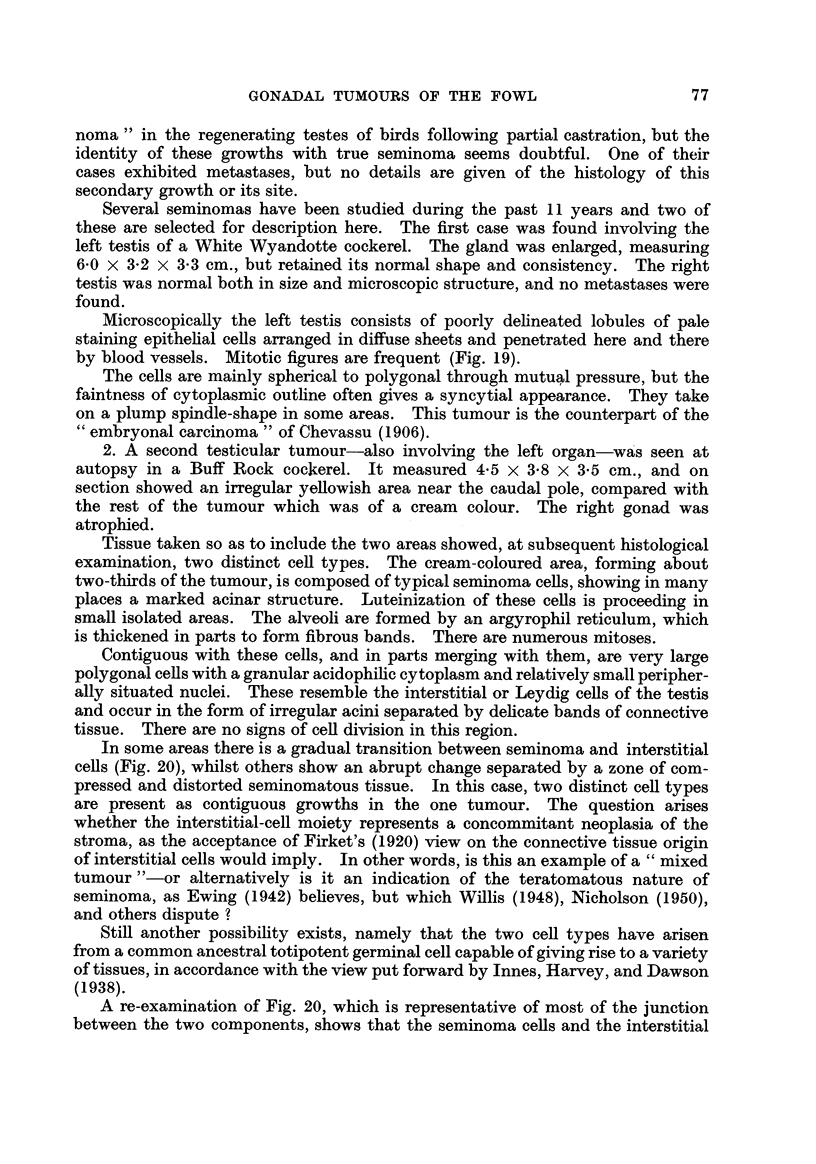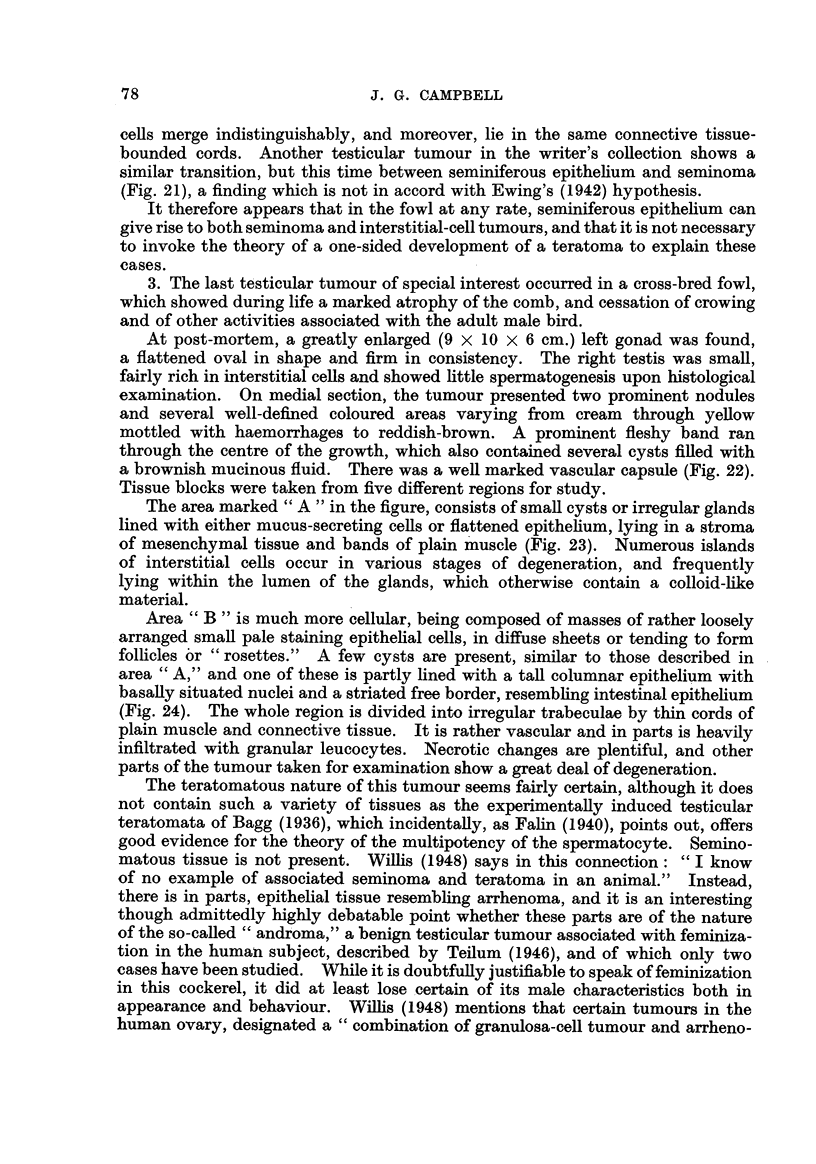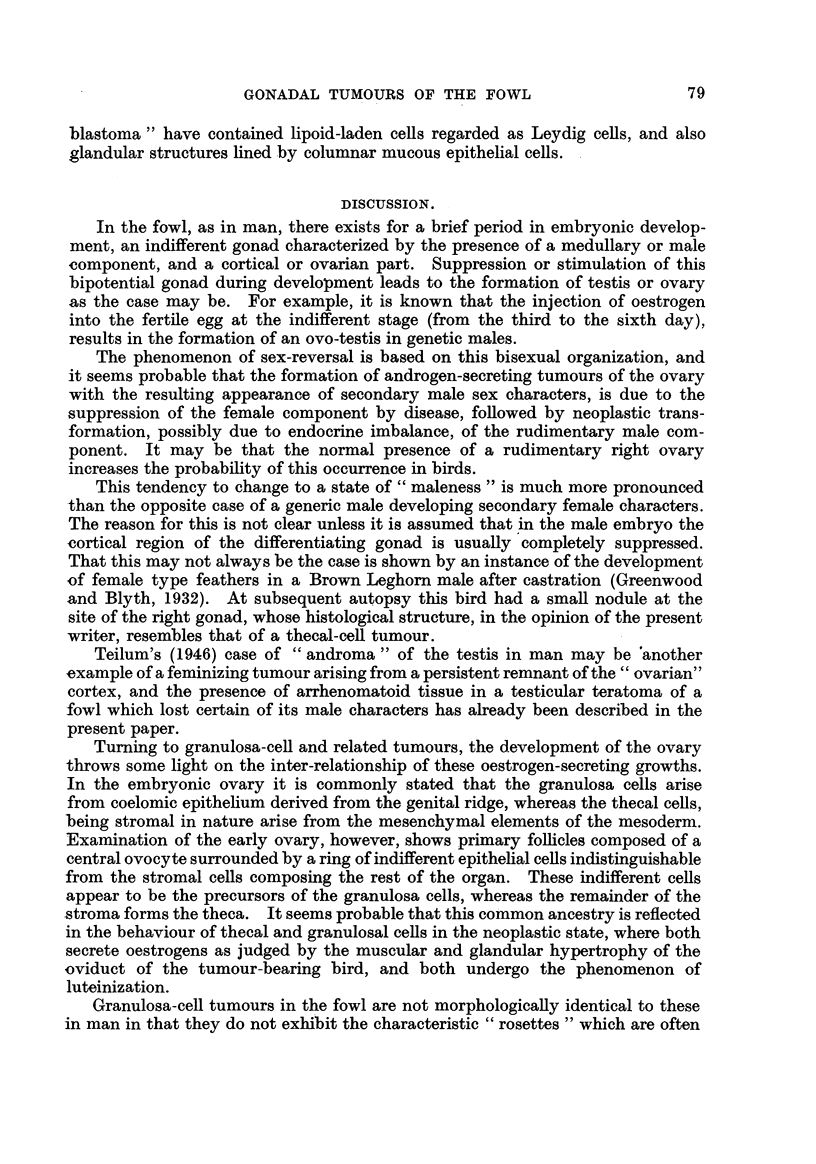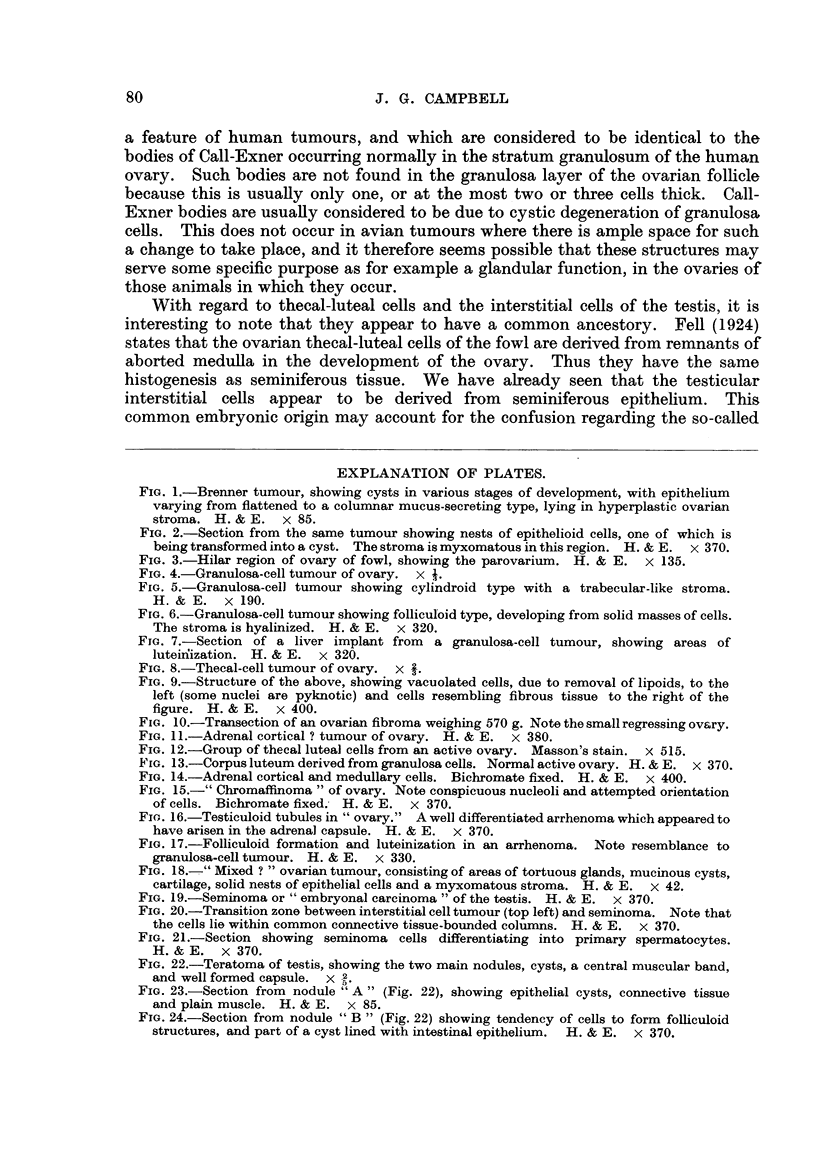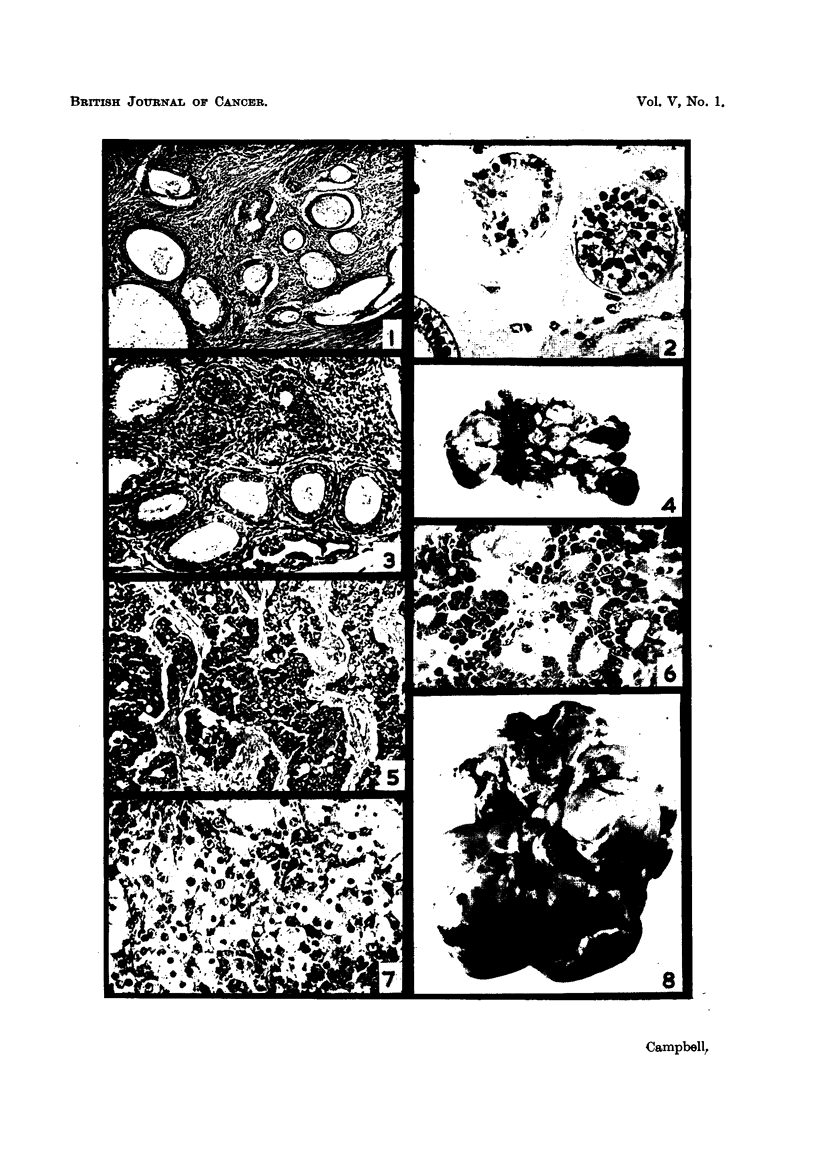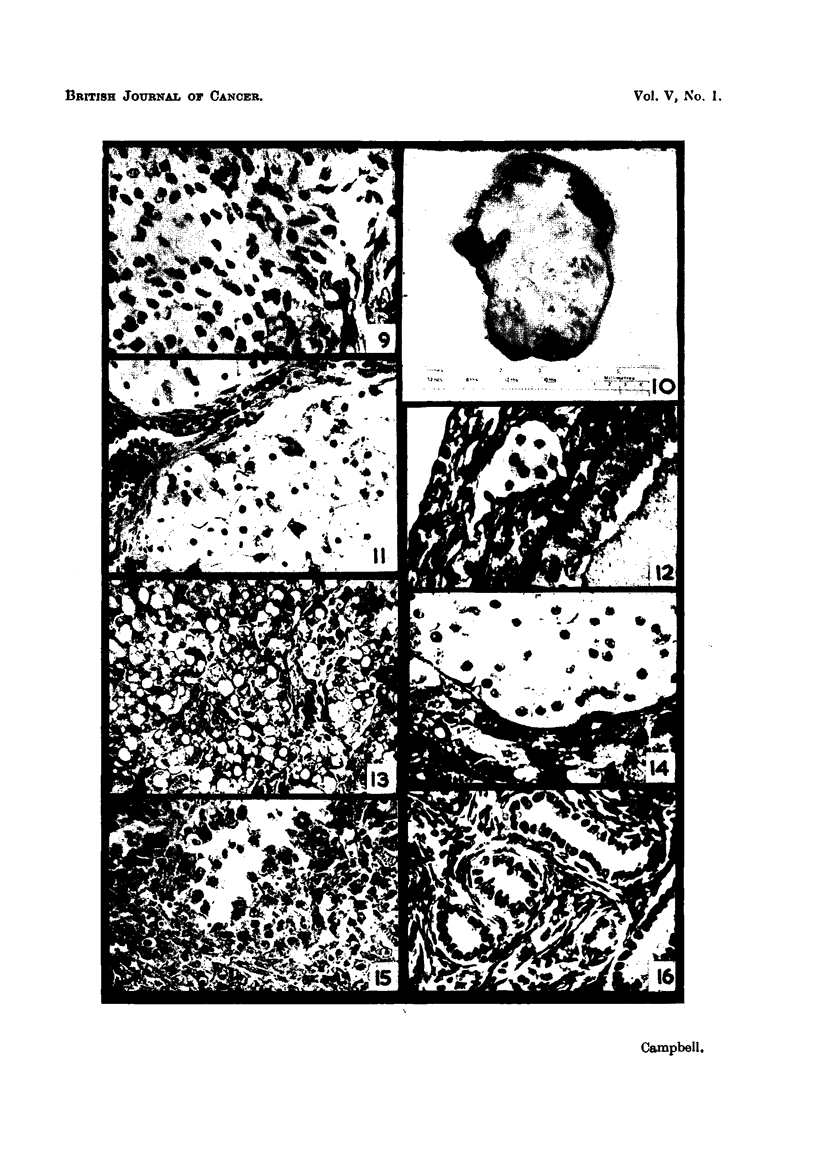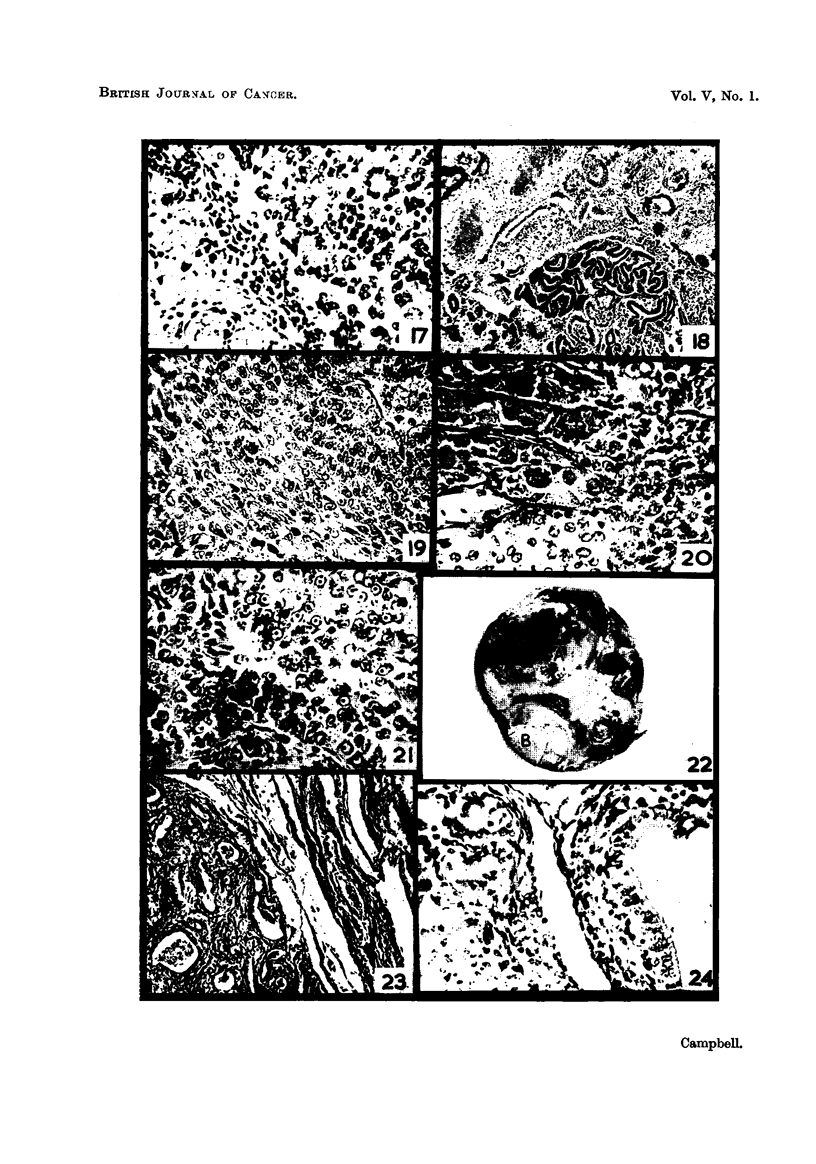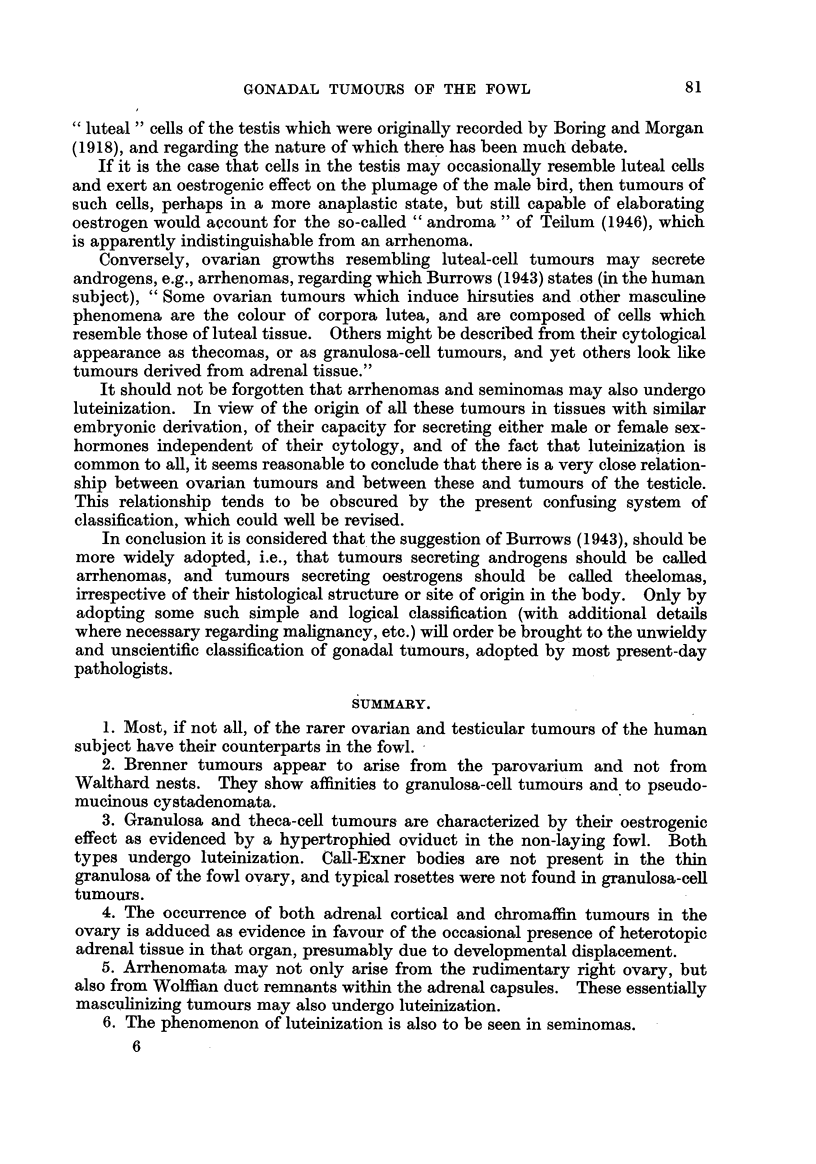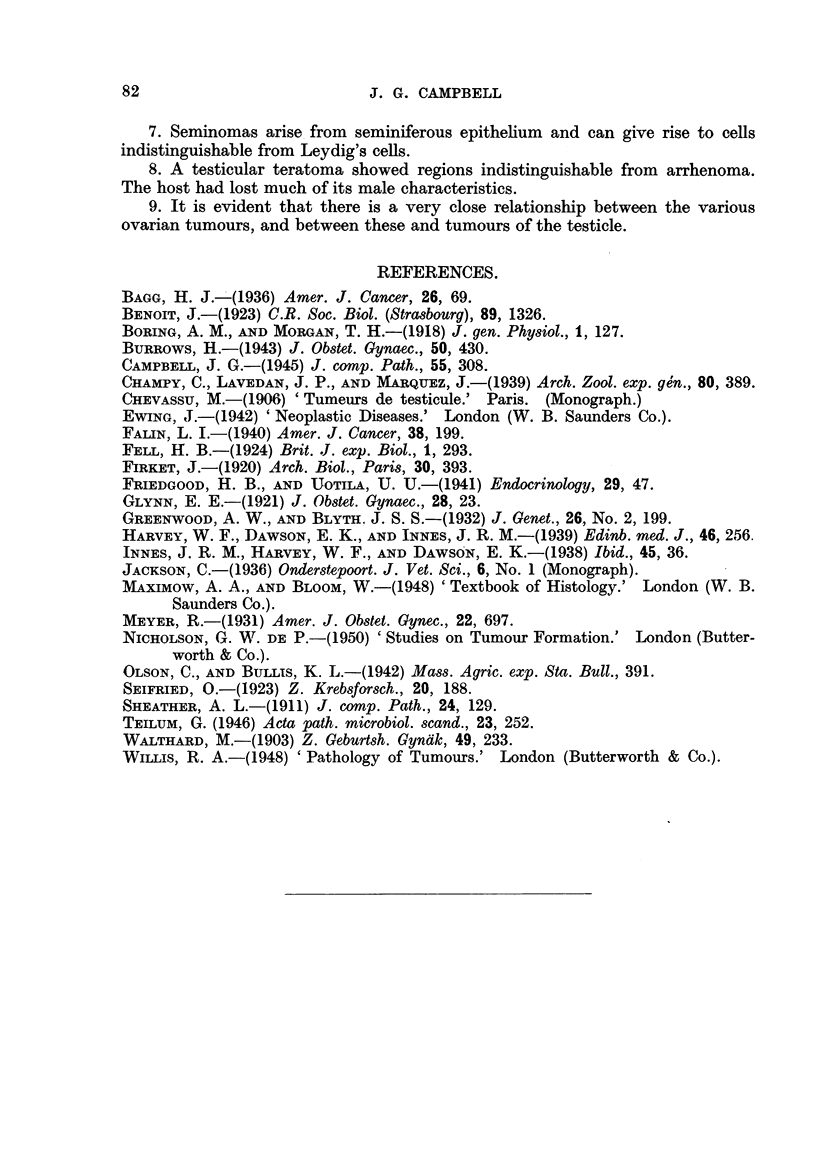# Some Unusual Gonadal Tumours of the Fowl

**DOI:** 10.1038/bjc.1951.7

**Published:** 1951-03

**Authors:** J. G. Campbell

## Abstract

**Images:**


### SOME UNUSUAL GONADAL TUMOURS OF THE FOWL.

J. G. CAMPBELL.

*From the Royal (Dick) Veterinary College, Edinburgh, and the Poultry Research Centre, King's Buildings, West Mains Road, Edinburgh.*

Received for publication February 5, 1951

NEOPLASMS involving the ovary are common in the fowl (Olson and Bullis, 1942 ; Campbell, 1945). The great majority fall into one or other of two categories, namely adenocarcinomatous growths in various stages of differentiation from the frankly anaplastic type to the sclerosing tumour, or, secondly, lymphomatous growths associated with the leucotic complex, e.g., lymphocytoma, fowl paralysis tumour, aleukaemic lymphoid leucosis, etc. Most of these lymphoid growths should not be classified as ovarian tumours, since in view of the generalized nature of the leucotic process it is usually impossible to be certain whether they are primary in that organ or not. They are mentioned simply because of the frequent reference in avian pathological literature to " lymphoid ovarian tumours."

In human pathology, besides the common adenocarcinomatous types of ovarian tumour, several other rarer forms are recognized as follows : Brenner tumour (oöphoroma folliculare) : granulosa-cell, thecal, and luteal tumours ; arrhenoblastomas ; dysgerminomas.

A survey of the literature dealing with neoplastic disease of the fowl shows the apparent extreme rarity of such tumours. Seifried (1923) recorded a Brenner tumour of the fowl and compared it with its human counterpart, and Friedgood and Uotila (1941) detailed 5 cases of ovarian " tumours " associated with virilism in the fowl. In two of these latter cases, tuberculosis of the ovary complicated the picture, and the remainder were cystic or contained small growths which appeared to be tumours in various stages of degeneration. Tentative diagnoses of arrhenoblastoma were made, but it was also suggested that they might be luteal-cell tumours or " hypernephromata." The only record of an ovarian teratoma in the fowl is by Jackson (1936).

With regard to the male bird, there appear to be no reports of spontaneous seminoma or interstitial-cell tumour, but several cases of spontaneous teratoma testis have been recorded (Sheather, 1911 ; Jackson, 1936 ; Olson and Bullis, 1942).

It seemed reasonable to believe that the absence of reports of the rarer forms of ovarian and testicular tumours was simply an indication of a lack of intensive study in this branch of comparative pathology. It was therefore determined to examine as many tumours arising in these sites as possible. To date approximately 2000 cases have been submitted to a thorough post-mortem and histological scrutiny, and, as was anticipated, several hitherto unrecorded types of tumour of the fowl gonads have been encountered.

It is desirable that an account of these should be put on record, not only because of their interest from the viewpoint of comparative oncology, but also because the study of such tumours may throw a light on the origin of their human counterparts, and may help to settle the much debated questions of inter-relationship and correlation between histological structure and endocrinological function.

### METHODS.

All the tumours described in this paper arose spontaneously. Some of the birds came to autopsy without any history. Others were obtained alive with a history of unusual behaviour such as the development of male traits in the hen, cessation of laying, enlarging abdomen, etc. At post-mortem a thorough search was made for metastases, and the possibility that the gonadal tumour was not the primary growth was ruled out as far as possible. Blocks of tissue were taken from many organs for histological examination, whether they showed visible abnormality or not. They were fixed in formal-saline, Susa or Helly, embedded in paraffin wax and cut at $8\mu$, and were stained by a variety of methods. Frozen sections were studied when the occasion permitted. Photographs of gross specimens were taken in many cases.

### PATHOLOGY.

### *Tumours of the Female Gonad.*

Tumours of the female gonads will be dealt with first.

(1) This tumour was encountered in a Rhode Island Red hen in its third year. Upon cessation of laying the bird was killed for table purposes, and the carcase was brought to the laboratory upon the same day.

The ovary was inactive, and a large part of it was replaced by a firm, faintly yellow and smoothly bosselated tumour $4{\cdot}0 \times 3{\cdot}5 \times 3{\cdot}8$ cm. in dimension. A few small cysts were visible on the surface, and a larger cyst filled with a brownish mucoid fluid occupied the caudal pole of the mass. The growth offered some resistance to the knife, and its cut surface showed pale yellow fibrous areas containing numerous small cysts and islands of pinkish tissue. There were no implantations in the abdominal cavity and no metastases were found.

Histologically a large part of the tumour consists of a stroma of interlacing bands and whorls of fibrous tissue, mostly dense, but having a looser texture in some places. There is a certain amount of plain muscle (characteristic of the avian ovary especially at the hilus) in one part of a section. Embedded in the dense fibrous tissue are numerous epithelial-lined cysts of varying size, some exhibiting a single layer of flattened cells, others lined by several layers, the innermost of which are typical columnar mucous secreting cells (Fig. 1). No cilia are detectable. The lumen of many cysts contains a granular eosinophilic material. Scattered between these structures are a few solid nests of undifferentiated epithelial cells with large pale nuclei. These show a tendency to develop a lumen (Fig. 2).

In view of its undoubted ovarian origin, its benign character and its strikingly similar structure to the Brenner tumour of the human subject, this case—the only one of its kind in the whole series of ovarian tumours so far examined—was classified as such. According to Willis (1948) it is probable that Brenner tumours arise in the human from nests of cells first described by Walthard in 1903. These may be found on the surface of the ovary, Fallopian tube or broad ligament. At the same time he points out that no extra-ovarian Brenner tumour has ever been reported, which, in view of the distribution of Walthard nests, raises the question of the validity of this etiological theory.

In an effort to establish whether comparable " nests " are present in the fowl, a considerable number of ovaries, oviducts and dorsal ligaments have been examined both by naked eye and histologically, with negative results. Such a finding of course does not rule out their existence in the fowl, but it does indicate that should other workers fail to find them, an alternative explanation will have to be considered. It may be relevant to indicate here that in the hilus of the fowl's ovary and in the oviduct ligament, a vestigial structure is to be found representing a rudiment of the genital part of the mesonephros and corresponding to the epididymis of the male. A comparison of this structure (Fig. 3) known as the parovarium, with the tumour described above shows a great similarity between the two. A comparable structure occurs in the human subject, and is well illustrated by Nicholson (1950).

(2) The next ovarian tumour to be considered is the granulosa-cell type, of which 4 cases have been studied in this series. They all occurred in adult hens, namely 2 Buff Rocks and 2 crossbreds. Unfortunately none of these was seen alive. In 2 cases there were extensive implantations from multiple ovarian tumours (Fig. 4) to the serous surface of the gut, oviduct, liver and kidney, and in one instance a metastatic growth was found in the lung. In the other 2 cases, the tumours were found at the site of the rudimentary right gonad, and one was associated with a small persistent cystic right oviduct. In both cases the left functional ovary appeared normal.

The gross appearance of these tumours varied somewhat. In general they were fairly firm, white, or with yellowish areas, sometimes cystic and occasionally haemorrhagic. It was interesting to note that in the first 2 cases mentioned above, although the ovary was not functional, the oviduct had the appearance and was nearly the size of an active organ.

Histologically, these tumours are characterized by a quite variable structure in different regions or even in different areas of the same section. One case, for example, shows mainly a " cylindroid " type of growth, with cords of polygonal epithelial cells with large clear nuclei and one or two prominent acidophilic nucleoli, lying in a fine connective tissue stroma (Fig. 5). A folliculoid formation is common in some areas, and the stroma between these groups of cells shows hyalinization (Fig. 6). Other cases show a more undifferentiated structure, but still a tendency to form " follicles," and in one case a very marked luteinization of the granulosa cells was present (Fig. 7). So noticeable is this feature in certain areas that a diagnosis of luteoma would be justifiable. " Typical " rosettes as seen in human tumours, characterized by a stellate arrangement of cells around a cystic space containing fluid or a degenerating cell, apparently do not occur in avian granulosa-cell tumours.

(3) One example of a theca-cell tumour was found at autopsy in a Buff Rock hen in her second year. It consisted of a yellowish irregular nodular mass measuring $5 \times 6 \times 4{\cdot}5$ cm. (Fig. 8). It was of a soft consistency and the exterior tended to be friable. On section, it was cream to yellowish in colour and displayed bands of tissue which had a tendency to radiate from the centre of the growth. The vascularity appeared to be very poor. The rest of the ovary seemed normal, with a few atretic follicles, and the oviduct was that of a bird in full lay. Histologically, this tumour is mainly composed of round oval or fusiform cells, with a fine chromatin network in the nucleus and a rather foamy cytoplasm (Fig. 9). Mitosis is extremely rare. In some regions, particularly near the periphery, these cells become polyhedral, with round nuclei and vacuolated cytoplasm. Frozen sections stained with Sudan IV show abundant lipoid material in such cells. The periphery of the growth is largely necrotic. Radiating bands of smooth muscle occur in the interior of the section.

Willis (1948) states that ovarian fibromata are probably the end result of a theca-cell tumour. In that case, the rarity of the latter in fowls may account for the equally rare occurrence of fibrous tissue tumours associated with this organ. As an instance of this the only case of ovarian fibroma encountered in this series is worth mentioning. It was found in a Rhode Island Red hen in her third year, and weighed 570 g. including the small inactive ovary (Fig. 10).

(4) Glynn (1921) states that there is no evidence (in the human subject) that heterotopic adrenal cortical tumours arise in the ovary. Willis (1948) agrees, and adds that no acceptable report exists showing the presence of accessory adrenal tissue in the ovary, and he concludes with Glynn that " these growths are all luteinized ovarian tumours." Later, however (p. 510), Willis admits the possibility of very rare adrenal cortical tumours primary in the ovary. Maximow and Bloom (1948) state that what are usually considered chromaffin cells (sympathicotrophic cells) are to be found at the hilus of the human ovary. These apparently have much in common with the medullary cells of the adrenal, although they cautiously conclude that until these cells have been shown to elaborate epinephrin, their relationship to adrenal medulla must remain unproven. The chromaffin reaction of such ovarian cells is unreliable but this also applies to the adrenal medullary cells, where the staining by chrome salts may vary from practically none to a very deep brown.

As far as can be ascertained there is no record of chromaffin cells or heterotopic adrenal cortical tissue in the avian ovary. That such cells may in fact be occasionally present seems to be implied by the following cases, and a thorough cytological examination of the region of the hilus should clear up this point.

The first of these tumours to be described occurred in a Brown Leghorn hen which was brought for examination after developing signs of masculinization. The bird was said to have ceased laying, commenced crowing and to have endeavoured to copulate with other hens in the run. The comb was decidedly male in character, and plumage changes were evident especially in the neck and tail.

Upon post-mortem, a rounded soft yellow-brown tumour measuring $3{\cdot}8 \times 3{\cdot}5 \times 2{\cdot}8$ cm. was found in the ovary, which was inactive. The oviduct was atrophied. Both adrenals were intact and were removed with other organs for examination.

Histologically the tumour has a distinct capsule. Numerous irregular trabeculae containing vessels arise from this and pass into the interior of the growth. Between these are solid islands of large polyhedral epithelial cells which are in turn divided into small irregular groups by fine connective tissue staining as for reticulum. The cytoplasm is eosinophilic and faintly granular. In places it is vacuolated. The nuclei are spherical and fairly constant in size, being situated usually in the centre of the cell. They have a finely scattered chromatin content and one or two inconspicuous nucleoli. There are no mitotic figures (Fig. 11).

This case is an excellent example of the pitfalls of diagnosis based upon purely morphological grounds, especially when applied to gonadal neoplasms. Superficially the tumour may be thought to resemble a luteoma, but the secretion of androgens weighs against this. It is of interest however to compare it with a small group of thecal luteal cells in the fowl's ovary (Fig. 12). A " corpus luteum " derived from the granulosa cells presents an entirely different picture (Fig. 13).

Other possible explanations are two in number. Firstly the growth may represent a heterotopic adrenal cortical tumour, which Ewing (1942) states to be highly masculinizing but otherwise difficult to differentiate from luteomas. The views of Willis (1948) on the subject have already been stated. Or secondly it may be a case of arrhenoma with such advanced luteinization (Fig. 16) that it bears no resemblance to " text-book " illustrations of that tumour.

It may be stated here that Meyer (1931) concludes that there is hardly any ovarian tumour pattern which cannot be reproduced in the arrhenomata. This view is substantially supported by Burrows (1943). If this is the case, then obviously ovarian tumours can no longer be classified according to their histology but only by their endocrine effects, if any, on the host.

What are we to conclude ? A comparison of the histology of this tumour with normal adrenal cortical tissue of the fowl shows a striking resemblance (Fig. 14). Silver preparations for reticulum also resemble the pattern found in the adrenal cortex. Measurements of thecal luteal cells in the ovary, adrenal cortical cells, and those in the tumour under discussion show that the last two have a similar range in size, whereas the thecal cells are smaller (thecal luteal cells 11·2$\mu$, nucleus 3·2$\mu$, adrenal cortical cells 16·0$\mu$, nucleus 4·8$\mu$, and tumour cells 19·2$\mu$, nucleus 4·8$\mu$, all measurements averages from fixed cells.)

It is now generally agreed that morphological resemblance does not necessarily imply physiological identity of function. Indeed Willis (1948) goes so far as to say " It would not be surprising if further research should show that the two classes of tumour (œstrogenic and androgenic) are really one, and both derived from the ovarian parenchyma." If this should prove to be the case, the naming of such tumours on morphological grounds cannot be justified, and they should be classified according to their endocrine activity.

This tumour was associated with virilism, and is therefore presumably a heterotopic adrenal cortical cell growth or an arrhenoma. The first view is favoured as being the more probable.

(5) The next case, though not a masculinizing growth, is worth recording for its unusual nature, and because its interpretation could mean that besides adrenal cortical cells, chromaffin cells resembling those of the adrenal medulla may occur in the fowl's ovary and may undergo neoplasia in common with other heterotopic tissues.

During the examination of a crossbred fowl which was found dead, a large intra-abdominal blood clot was found proceeding from a rupture in the substance of a smoothly lobulated brownish-yellow tumour of soft consistency, measuring 6·5 × 5 × 4·3 cm. and occupying the site of the left (functional) ovary. It was freely movable, being only attached at the hilus, and was therefore easily dissected out. Numerous small cream-coloured tumours were scattered in the substance of the liver, spleen and kidneys. The adrenals seemed normal and this was subsequently confirmed by histological study.

Section of the gross specimen showed it to have a richly vascular outer zone and an irregular inner zone, of a spongy brown character. The periphery of the growth tended to strip off in layers of what appeared to be necrotic tissue.

It is fortunate that the several blocks of tissue taken from various regions were fixed in Helly's fluid, as well as other fixatives, since it is possible that the unusual nature of the tumour would have been largely missed, had it not been chromed.

Histological examination of the outer zone shows it to be necrotic in some parts. The healthy remainder is composed of connective tissue cells with spherical to spindle-shaped nuclei, and eosinophilic granular cytoplasm, surrounding aggregations of large polyhedral cells with abundant finely granular eosinophilic cytoplasm, large nuclei and very prominent nucleoli (nuclear-nucleolar ratio 1:3 to 1:5).

An abundant infiltration with eosinophils is present in many parts of the small epithelial-cell moiety.

A well-marked but thin layer of connective tissue separates this outer zone from the inner, which is composed of small lobules of cells separated from each other by fine trabeculae of connective tissue. The cells composing these groups are large, with giant nucleoli similar to those mentioned above, and have a brownish-yellow cytoplasm in bichromate fixed haematoxylin and eosin preparations. They are frequently arranged in palisade formation at the periphery of the lobule, and their disposition and appearance in the interior of the groups is strikingly similar (save in size of nucleoli) to the cells composing the avian adrenal medulla (Fig. 15 and compare with previous figure). Areas of necrosis and spaces filled with fibrinous material are common in the inner zone, which is also very vascular.

Other regions of the tumour, and the metastatic lesions in the liver, spleen, and kidneys, consist mainly of polyhedral cells with large vacuolated nuclei and prominent nucleoli, tending to palisade small acini whose basement membrane is composed of reticulum fibrils. The cytoplasm of these cells is finely granular and heterophilic, and mitotic figures are common. At the edge of one series of sections, the only indication of ovarian origin is the presence of a few follicles. Bands of smooth muscle are scattered throughout the section.

The presence of such large numbers of cells showing a distinct chromaffin reaction justifies a diagnosis of chromaffinoma or paraganglioma. One hesitates to speak of a phaeochromocytoma of the ovary since no record of such tumour is in existence, but the term is really synonymous. The nature of the remaining cells of this tumour is debatable. Some are similar to the chromaffin cells, except there is no brown staining of the cytoplasm, and could conceivably be young forms. Others are mesenchymal in nature and are strongly reminiscent of thecal cells, a view which is supported by the fact that isolated groups of such cells are seen to be undergoing luteinization; and finally the polyhedral cells palisading the small acini which make up the rest of the tumour and metastatic growths, resemble granulosa-cells. This tumour, then, is a complex in which chromaffin, theca and granulosa-cells all occur, the latter metastasizing to the liver, spleen and kidneys.

The ovarian tumour just described apparently had no noticeable effect on the behaviour of the bird. In the human subject phaeochromacytomas are associated with raised blood pressure. It may not be entirely coincidence that this bird died as a result of an internal haemorrhage.

(6) The following case is another example of a masculinizing tumour. It occurred in a Brown Leghorn " hen " at this Centre, and was originally studied by Drs. Blyth and Carr, who supplied the following details.

The bird appeared to be a male when killed, although originally it had a normal female appearance. It was used to propagate the Duran-Reynals filterable sarcoma. *Post-mortem* examination showed a small tumour at the site of the left ovary, normal in shape for that organ, but with no macroscopically-visible ova, measuring 1·2 × 0·7 cm. It was closely adherent to the left adrenal gland, and was sectioned with it. This tumour is composed of well-developed testiculoid elements lying in a stroma of undifferentiated spindle-shaped cells (Fig. 16). Many solid cords of epithelioid cells lie just below the germinal epithelium investing the surface. The growth appears to have arisen within the adrenal capsule.

Another small (0·5 × 0·2 cm.) tumour was found in the site of the rudimentary right gonad and closely adherent to the dorsal wall. This shows not only testiculoid elements, but also a considerable area composed of whorls and strands of rapidly dividing mesenchymatous cells, presumably a metastasis from the experimentally transplanted sarcoma.

The histology of both gonadal growths resembles Ewing's (1942) Fig. 316, illustrating a " testicular adenoma of the ovary," a well-differentiated tumour belonging to the arrhenoma group. The nests of cells below the germinal epithelium resemble the primitive sex cords of the indifferent embryonic gonad. This tumour illustrates well the bisexuality of the embryonic fowl's ovary. The *rete ovarii* formed from the primitive sex cords is a homologue of the testis and as is well known can develop into one following left ovariectomy of the hen, where the right gonad hypertrophies and differentiates even to the extent of producing spermatozoa (Benoit, 1923).

Another tumour of the ovary associated with masculinization in a Light Sussex hen is shown in Fig. 17. Folliculoid formation and luteinization is apparent, and the structure is comparable to a granulosa-cell tumour. In fact, this diagnosis would be justifiable on histological grounds alone, but has to be modified in view of the androgenic effects on the host.

It would appear that the rudimentary right ovary is not the only place where in theory at any rate, testiculoid structures could arise in the intact bird. Champy, Lavedan and Marquez (1939) state that the adrenals of either male or female castrates in the fowl frequently contain " regenerating gonadal cells." Birds' adrenals constantly contain Wolffian duct remnants in the capsule on the ventral internal aspect. These slowly hypertrophy following castration to form an epididymis-like structure, whilst some form epithelial tubules similar to those found in the right ovarian rudiment subsequent to castration. Spermatogenesis has not been seen in the adrenal capsule however. There does not seem to be any record of spontaneous arrhenoma arising in the adrenal capsule of the fowl, but it seems possible that the foregoing is a case in point.

(7) The last of the tumours of the female gonad to be described was originally considered to be a teratoma. It is unfortunate that no comparison can be made of this case with the only comparable ovarian tumour of the fowl on record (Jackson, 1936), since apart from stating its didermic character, no other details were given, and the single photograph is of the gross specimen.

The present case occurred in a Brown Leghorn hen aged about 3 years, at the Poultry Research Centre. The bird had ascites and ovarian tumour was diagnosed. Red blood cells, macrophages and epithelial cells were seen in a smear of the centrifuged deposit from the brown turbid fluid, 140 ml. of which was withdrawn from the abdomen. After death 4 days later, an ovarian tumour was found measuring 5 × 4 × 4 cm. It was mottled pink, brownish and cream, of firm consistency and mucinous on section. Other growths, pinkish and soft, were attached to the wall of the oviduct, and to the serous investment of the duodenum, pancreas and liver.

Histologically the ovarian tumour is the only one to show complexity of structure. Immediately below a thin capsule there are nests of large pale staining epithelial cells, some of which have differentiated into cysts of varying size, lined with one or several layers of columnar epithelium. In many cases these cells are actively secreting mucus. Large areas of the tumour consist of a vascular myxo-chondromatous matrix showing all stages of development of hyaline cartilage from a myxomatous tissue to irregular islands of well developed cartilage. Complicated folded, branched and invaginated epithelial tubules, and clumps of actively dividing cells often forming folliculoid structures complete the picture (Fig. 18). The implants on the viscera show a typical folliculoid type of granulosa-cell tumour.

As with many ovarian tumours, this case is not easy to classify. At first sight two embryonic layers appear to be involved, namely mesoderm and ectoderm. The latter may be represented by the cyst epithelium forming mucinous glands, and possibly as the complex epithelial tubular structures. The remainder of the tumour is mesodermal in origin. If this is the case the presence of these derivatives of two germinal layers presumably puts it in the same category as Jackson's (1936) "didermic teratoma." Willis (1948) defines a teratoma as "a true tumour or neoplasm composed of multiple tissues of kinds foreign to the part in which it arises." The presence of cartilage can be explained by the conversion of connective tissue normally present in the ovary, to cartilage by mucoid degeneration and hyalinization. At first sight the mucus-secreting epithelial cells of the cysts seem to satisfy the definition of Willis, but such structures have been described by Harvey, Dawson and Innes (1939), in Brenner-cell tumours in the human subject, and therefore presumably arise from cells indigenous to the ovary. Also it should be noted that the same authors express the view that Brenner and granulosa-cell tumours are intimately related, an opinion also held by Willis (1948).

Is a diagnosis of teratoma justifiable ? Nicholson (1950) stresses the necessity of the identification of tissues representing all three germinal layers before a diagnosis of teratoma is made. It has been shown that in this case it is doubtful whether even two layers are in fact represented, and so the view is taken that we are here dealing with a neoplasm demonstrating the close relationship between Brenner tumour, and pseudomucinous cystadenoma, in which metaplasia of myxomatous tissue has given rise to hyaline cartilage, whilst other elements have differentiated into a mature type of epithelium secreting mucus, and an embryonic moiety represented by the granulosa-cell elements has spread by implantation to organs in the abdominal cavity.

*Tumours of the Male Gonad.*

1. It seems extraordinary that no report exists of spontaneous seminoma in the fowl. Champy, Lavedan and Marquez (1939) describe 4 cases of "seminoma " in the regenerating testes of birds following partial castration, but the identity of these growths with true seminoma seems doubtful. One of their cases exhibited metastases, but no details are given of the histology of this secondary growth or its site.

Several seminomas have been studied during the past 11 years and two of these are selected for description here. The first case was found involving the left testis of a White Wyandotte cockerel. The gland was enlarged, measuring 6·0 × 3·2 × 3·3 cm., but retained its normal shape and consistency. The right testis was normal both in size and microscopic structure, and no metastases were found.

Microscopically the left testis consists of poorly delineated lobules of pale staining epithelial cells arranged in diffuse sheets and penetrated here and there by blood vessels. Mitotic figures are frequent (Fig. 19).

The cells are mainly spherical to polygonal through mutual pressure, but the faintness of cytoplasmic outline often gives a syncytial appearance. They take on a plump spindle-shape in some areas. This tumour is the counterpart of the " embryonal carcinoma " of Chevassu (1906).

2. A second testicular tumour—also involving the left organ—was seen at autopsy in a Buff Rock cockerel. It measured 4·5 × 3·8 × 3·5 cm., and on section showed an irregular yellowish area near the caudal pole, compared with the rest of the tumour which was of a cream colour. The right gonad was atrophied.

Tissue taken so as to include the two areas showed, at subsequent histological examination, two distinct cell types. The cream-coloured area, forming about two-thirds of the tumour, is composed of typical seminoma cells, showing in many places a marked acinar structure. Luteinization of these cells is proceeding in small isolated areas. The alveoli are formed by an argyrophil reticulum, which is thickened in parts to form fibrous bands. There are numerous mitoses.

Contiguous with these cells, and in parts merging with them, are very large polygonal cells with a granular acidophilic cytoplasm and relatively small peripherally situated nuclei. These resemble the interstitial or Leydig cells of the testis and occur in the form of irregular acini separated by delicate bands of connective tissue. There are no signs of cell division in this region.

In some areas there is a gradual transition between seminoma and interstitial cells (Fig. 20), whilst others show an abrupt change separated by a zone of compressed and distorted seminomatous tissue. In this case, two distinct cell types are present as contiguous growths in the one tumour. The question arises whether the interstitial-cell moiety represents a concommitant neoplasia of the stroma, as the acceptance of Firket's (1920) view on the connective tissue origin of interstitial cells would imply. In other words, is this an example of a " mixed tumour "—or alternatively is it an indication of the teratomatous nature of seminoma, as Ewing (1942) believes, but which Willis (1948), Nicholson (1950), and others dispute ?

Still another possibility exists, namely that the two cell types have arisen from a common ancestral totipotent germinal cell capable of giving rise to a variety of tissues, in accordance with the view put forward by Innes, Harvey, and Dawson (1938).

A re-examination of Fig. 20, which is representative of most of the junction between the two components, shows that the seminoma cells and the interstitial cells merge indistinguishably, and moreover, lie in the same connective tissue-bounded cords. Another testicular tumour in the writer's collection shows a similar transition, but this time between seminiferous epithelium and seminoma (Fig. 21), a finding which is not in accord with Ewing's (1942) hypothesis.

It therefore appears that in the fowl at any rate, seminiferous epithelium can give rise to both seminoma and interstitial-cell tumours, and that it is not necessary to invoke the theory of a one-sided development of a teratoma to explain these cases.

3. The last testicular tumour of special interest occurred in a cross-bred fowl, which showed during life a marked atrophy of the comb, and cessation of crowing and of other activities associated with the adult male bird.

At post-mortem, a greatly enlarged (9 × 10 × 6 cm.) left gonad was found, a flattened oval in shape and firm in consistency. The right testis was small, fairly rich in interstitial cells and showed little spermatogenesis upon histological examination. On medial section, the tumour presented two prominent nodules and several well-defined coloured areas varying from cream through yellow mottled with haemorrhages to reddish-brown. A prominent fleshy band ran through the centre of the growth, which also contained several cysts filled with a brownish mucinous fluid. There was a well marked vascular capsule (Fig. 22). Tissue blocks were taken from five different regions for study.

The area marked " A " in the figure, consists of small cysts or irregular glands lined with either mucus-secreting cells or flattened epithelium, lying in a stroma of mesenchymal tissue and bands of plain muscle (Fig. 23). Numerous islands of interstitial cells occur in various stages of degeneration, and frequently lying within the lumen of the glands, which otherwise contain a colloid-like material.

Area " B " is much more cellular, being composed of masses of rather loosely arranged small pale staining epithelial cells, in diffuse sheets or tending to form follicles or " rosettes." A few cysts are present, similar to those described in area " A," and one of these is partly lined with a tall columnar epithelium with basally situated nuclei and a striated free border, resembling intestinal epithelium (Fig. 24). The whole region is divided into irregular trabeculae by thin cords of plain muscle and connective tissue. It is rather vascular and in parts is heavily infiltrated with granular leucocytes. Necrotic changes are plentiful, and other parts of the tumour taken for examination show a great deal of degeneration.

The teratomatous nature of this tumour seems fairly certain, although it does not contain such a variety of tissues as the experimentally induced testicular teratomata of Bagg (1936), which incidentally, as Falin (1940), points out, offers good evidence for the theory of the multipotency of the spermatocyte. Seminomatous tissue is not present. Willis (1948) says in this connection : " I know of no example of associated seminoma and teratoma in an animal." Instead, there is in parts, epithelial tissue resembling arrhenoma, and it is an interesting though admittedly highly debatable point whether these parts are of the nature of the so-called " androma," a benign testicular tumour associated with feminization in the human subject, described by Teilum (1946), and of which only two cases have been studied. While it is doubtfully justifiable to speak of feminization in this cockerel, it did at least lose certain of its male characteristics both in appearance and behaviour. Willis (1948) mentions that certain tumours in the human ovary, designated a " combination of granulosa-cell tumour and arrhenoblastoma " have contained lipoid-laden cells regarded as Leydig cells, and also glandular structures lined by columnar mucous epithelial cells.

### DISCUSSION.

In the fowl, as in man, there exists for a brief period in embryonic development, an indifferent gonad characterized by the presence of a medullary or male component, and a cortical or ovarian part. Suppression or stimulation of this bipotential gonad during development leads to the formation of testis or ovary as the case may be. For example, it is known that the injection of oestrogen into the fertile egg at the indifferent stage (from the third to the sixth day), results in the formation of an ovo-testis in genetic males.

The phenomenon of sex-reversal is based on this bisexual organization, and it seems probable that the formation of androgen-secreting tumours of the ovary with the resulting appearance of secondary male sex characters, is due to the suppression of the female component by disease, followed by neoplastic transformation, possibly due to endocrine imbalance, of the rudimentary male component. It may be that the normal presence of a rudimentary right ovary increases the probability of this occurrence in birds.

This tendency to change to a state of " maleness " is much more pronounced than the opposite case of a generic male developing secondary female characters. The reason for this is not clear unless it is assumed that in the male embryo the cortical region of the differentiating gonad is usually completely suppressed. That this may not always be the case is shown by an instance of the development of female type feathers in a Brown Leghorn male after castration (Greenwood and Blyth, 1932). At subsequent autopsy this bird had a small nodule at the site of the right gonad, whose histological structure, in the opinion of the present writer, resembles that of a thecal-cell tumour.

Teilum's (1946) case of " androma " of the testis in man may be another example of a feminizing tumour arising from a persistent remnant of the " ovarian" cortex, and the presence of arrhenomatoid tissue in a testicular teratoma of a fowl which lost certain of its male characters has already been described in the present paper.

Turning to granulosa-cell and related tumours, the development of the ovary throws some light on the inter-relationship of these oestrogen-secreting growths. In the embryonic ovary it is commonly stated that the granulosa cells arise from coelomic epithelium derived from the genital ridge, whereas the thecal cells, being stromal in nature arise from the mesenchymal elements of the mesoderm. Examination of the early ovary, however, shows primary follicles composed of a central ovocyte surrounded by a ring of indifferent epithelial cells indistinguishable from the stromal cells composing the rest of the organ. These indifferent cells appear to be the precursors of the granulosa cells, whereas the remainder of the stroma forms the theca. It seems probable that this common ancestry is reflected in the behaviour of thecal and granulosal cells in the neoplastic state, where both secrete oestrogens as judged by the muscular and glandular hypertrophy of the oviduct of the tumour-bearing bird, and both undergo the phenomenon of luteinization.

Granulosa-cell tumours in the fowl are not morphologically identical to these in man in that they do not exhibit the characteristic " rosettes " which are often a feature of human tumours, and which are considered to be identical to the bodies of Call-Exner occurring normally in the stratum granulosum of the human ovary. Such bodies are not found in the granulosa layer of the ovarian follicle because this is usually only one, or at the most two or three cells thick. Call-Exner bodies are usually considered to be due to cystic degeneration of granulosa cells. This does not occur in avian tumours where there is ample space for such a change to take place, and it therefore seems possible that these structures may serve some specific purpose as for example a glandular function, in the ovaries of those animals in which they occur.

With regard to thecal-luteal cells and the interstitial cells of the testis, it is interesting to note that they appear to have a common ancestory. Fell (1924) states that the ovarian thecal-luteal cells of the fowl are derived from remnants of aborted medulla in the development of the ovary. Thus they have the same histogenesis as seminiferous tissue. We have already seen that the testicular interstitial cells appear to be derived from seminiferous epithelium. This common embryonic origin may account for the confusion regarding the so-called

---

### EXPLANATION OF PLATES.

FIG. 1.—Brenner tumour, showing cysts in various stages of development, with epithelium varying from flattened to a columnar mucus-secreting type, lying in hyperplastic ovarian stroma. H. & E. × 85.

FIG. 2.—Section from the same tumour showing nests of epithelioid cells, one of which is being transformed into a cyst. The stroma is myxomatous in this region. H. & E. × 370.

FIG. 3.—Hilar region of ovary of fowl, showing the parovarium. H. & E. × 135.

FIG. 4.—Granulosa-cell tumour of ovary. × $\frac{1}{3}$.

FIG. 5.—Granulosa-cell tumour showing cylindroid type with a trabecular-like stroma. H. & E. × 190.

FIG. 6.—Granulosa-cell tumour showing folliculoid type, developing from solid masses of cells. The stroma is hyalinized. H. & E. × 320.

FIG. 7.—Section of a liver implant from a granulosa-cell tumour, showing areas of luteinization. H. & E. × 320.

FIG. 8.—Thecal-cell tumour of ovary. × $\frac{2}{3}$.

FIG. 9.—Structure of the above, showing vacuolated cells, due to removal of lipoids, to the left (some nuclei are pyknotic) and cells resembling fibrous tissue to the right of the figure. H. & E. × 400.

FIG. 10.—Transection of an ovarian fibroma weighing 570 g. Note the small regressing ovary.

FIG. 11.—Adrenal cortical ? tumour of ovary. H. & E. × 380.

FIG. 12.—Group of thecal luteal cells from an active ovary. Masson's stain. × 515.

FIG. 13.—Corpus luteum derived from granulosa cells. Normal active ovary. H. & E. × 370.

FIG. 14.—Adrenal cortical and medullary cells. Bichromate fixed. H. & E. × 400.

FIG. 15.—" Chromaffinoma " of ovary. Note conspicuous nucleoli and attempted orientation of cells. Bichromate fixed. H. & E. × 370.

FIG. 16.—Testiculoid tubules in " ovary." A well differentiated arrhenoma which appeared to have arisen in the adrenal capsule. H. & E. × 370.

FIG. 17.—Folliculoid formation and luteinization in an arrhenoma. Note resemblance to granulosa-cell tumour. H. & E. × 330.

FIG. 18.—" Mixed ? " ovarian tumour, consisting of areas of tortuous glands, mucinous cysts, cartilage, solid nests of epithelial cells and a myxomatous stroma. H. & E. × 42.

FIG. 19.—Seminoma or " embryonal carcinoma " of the testis. H. & E. × 370.

FIG. 20.—Transition zone between interstitial cell tumour (top left) and seminoma. Note that the cells lie within common connective tissue-bounded columns. H. & E. × 370.

FIG. 21.—Section showing seminoma cells differentiating into primary spermatocytes. H. & E. × 370.

FIG. 22.—Teratoma of testis, showing the two main nodules, cysts, a central muscular band, and well formed capsule. × $\frac{2}{5}$.

FIG. 23.—Section from nodule " A " (Fig. 22), showing epithelial cysts, connective tissue and plain muscle. H. & E. × 85.

FIG. 24.—Section from nodule " B " (Fig. 22) showing tendency of cells to form folliculoid structures, and part of a cyst lined with intestinal epithelium. H. & E. × 370.

Campbell.

9
Inches
32nds
8ths
12 ths
10ths
Millimetres
10
11
12
13
14
15
16

Campbell.

Campbell.

" luteal " cells of the testis which were originally recorded by Boring and Morgan (1918), and regarding the nature of which there has been much debate.

If it is the case that cells in the testis may occasionally resemble luteal cells and exert an oestrogenic effect on the plumage of the male bird, then tumours of such cells, perhaps in a more anaplastic state, but still capable of elaborating oestrogen would account for the so-called " androma " of Teilum (1946), which is apparently indistinguishable from an arrhenoma.

Conversely, ovarian growths resembling luteal-cell tumours may secrete androgens, e.g., arrhenomas, regarding which Burrows (1943) states (in the human subject), " Some ovarian tumours which induce hirsuties and other masculine phenomena are the colour of corpora lutea, and are composed of cells which resemble those of luteal tissue. Others might be described from their cytological appearance as thecomas, or as granulosa-cell tumours, and yet others look like tumours derived from adrenal tissue."

It should not be forgotten that arrhenomas and seminomas may also undergo luteinization. In view of the origin of all these tumours in tissues with similar embryonic derivation, of their capacity for secreting either male or female sex-hormones independent of their cytology, and of the fact that luteinization is common to all, it seems reasonable to conclude that there is a very close relationship between ovarian tumours and between these and tumours of the testicle. This relationship tends to be obscured by the present confusing system of classification, which could well be revised.

In conclusion it is considered that the suggestion of Burrows (1943), should be more widely adopted, i.e., that tumours secreting androgens should be called arrhenomas, and tumours secreting oestrogens should be called theelomas, irrespective of their histological structure or site of origin in the body. Only by adopting some such simple and logical classification (with additional details where necessary regarding malignancy, etc.) will order be brought to the unwieldy and unscientific classification of gonadal tumours, adopted by most present-day pathologists.

### SUMMARY.

1. Most, if not all, of the rarer ovarian and testicular tumours of the human subject have their counterparts in the fowl.

2. Brenner tumours appear to arise from the parovarium and not from Walthard nests. They show affinities to granulosa-cell tumours and to pseudo-mucinous cystadenomata.

3. Granulosa and theca-cell tumours are characterized by their oestrogenic effect as evidenced by a hypertrophied oviduct in the non-laying fowl. Both types undergo luteinization. Call-Exner bodies are not present in the thin granulosa of the fowl ovary, and typical rosettes were not found in granulosa-cell tumours.

4. The occurrence of both adrenal cortical and chromaffin tumours in the ovary is adduced as evidence in favour of the occasional presence of heterotopic adrenal tissue in that organ, presumably due to developmental displacement.

5. Arrhenomata may not only arise from the rudimentary right ovary, but also from Wolffian duct remnants within the adrenal capsules. These essentially masculinizing tumours may also undergo luteinization.

6. The phenomenon of luteinization is also to be seen in seminomas.

7. Seminomas arise from seminiferous epithelium and can give rise to cells indistinguishable from Leydig's cells.

8. A testicular teratoma showed regions indistinguishable from arrhenoma. The host had lost much of its male characteristics.

9. It is evident that there is a very close relationship between the various ovarian tumours, and between these and tumours of the testicle.

### REFERENCES.

Bagg, H. J.—(1936) *Amer. J. Cancer*, **26**, 69.

Benoit, J.—(1923) *C.R. Soc. Biol.* (*Strasbourg*), **89**, 1326.

Boring, A. M., and Morgan, T. H.—(1918) *J. gen. Physiol.*, **1**, 127.

Burrows, H.—(1943) *J. Obstet. Gynaec.*, **50**, 430.

Campbell, J. G.—(1945) *J. comp. Path.*, **55**, 308.

Champy, C., Lavedan, J. P., and Marquez, J.—(1939) *Arch. Zool. exp. gén.*, **80**, 389.

Chevassu, M.—(1906) 'Tumeurs de testicule.' Paris. (Monograph.)

Ewing, J.—(1942) 'Neoplastic Diseases.' London (W. B. Saunders Co.).

Falin, L. I.—(1940) *Amer. J. Cancer*, **38**, 199.

Fell, H. B.—(1924) *Brit. J. exp. Biol.*, **1**, 293.

Firket, J.—(1920) *Arch. Biol., Paris*, **30**, 393.

Friedgood, H. B., and Uotila, U. U.—(1941) *Endocrinology*, **29**, 47.

Glynn, E. E.—(1921) *J. Obstet. Gynaec.*, **28**, 23.

Greenwood, A. W., and Blyth. J. S. S.—(1932) *J. Genet.*, **26**, No. 2, 199.

Harvey, W. F., Dawson, E. K., and Innes, J. R. M.—(1939) *Edinb. med. J.*, **46**, 256.

Innes, J. R. M., Harvey, W. F., and Dawson, E. K.—(1938) *Ibid.*, **45**, 36.

Jackson, C.—(1936) *Onderstepoort. J. Vet. Sci.*, **6**, No. 1 (Monograph).

Maximow, A. A., and Bloom, W.—(1948) 'Textbook of Histology.' London (W. B. Saunders Co.).

Meyer, R.—(1931) *Amer. J. Obstet. Gynec.*, **22**, 697.

Nicholson, G. W. de P.—(1950) 'Studies on Tumour Formation.' London (Butterworth & Co.).

Olson, C., and Bullis, K. L.—(1942) *Mass. Agric. exp. Sta. Bull.*, 391.

Seifried, O.—(1923) *Z. Krebsforsch.*, **20**, 188.

Sheather, A. L.—(1911) *J. comp. Path.*, **24**, 129.

Teilum, G. (1946) *Acta path. microbiol. scand.*, **23**, 252.

Walthard, M.—(1903) *Z. Geburtsh. Gynäk*, **49**, 233.

Willis, R. A.—(1948) 'Pathology of Tumours.' London (Butterworth & Co.).